# Multidrug resistance among uropathogenic clonal group A *E. Coli* isolates from Pakistani women with uncomplicated urinary tract infections

**DOI:** 10.1186/s12866-024-03221-8

**Published:** 2024-03-07

**Authors:** Ayesha Khan, Viqar Sayeed Saraf, Fariha Siddiqui, Tahira Batool, Zobia Noreen, Sundus Javed, Aftab Ahmad, Wadi B. Alonazi, Muhammad Ibrahim, Sandra Pucciarelli, Habib Bokhari

**Affiliations:** 1 Microbiology and Public Health Laboratory, Department of Biosciences, COMSATS University Islamabad, Islamabad, Pakistan; 2 Health Services Academy, Opposite NIH, Islamabad, Pakistan; 3 Department of Biosciences, Shifa Tameer e Millat University, Islamabad, Pakistan; 4 Department of Microbiology, Kohsar University Murree, Rawalpindi, Punjab Pakistan; 5 Health Administration Department, College of Business Administration, King Saud University, Riyadh, Saudi Arabia; 6 Department of Biosciences, COMSATS University Islamabad, Sahiwal Campus, Sahiwal, Pakistan; 7 School of Biosciences and Veterinary Medicine, University of Camerino, via Gentile III da Varano, Camerino, 62032 Italy

**Keywords:** Urinary tract infections, Low-income group, Premenopausal women, *E. Coli*, Phylotypes, *Galleria* infection model

## Abstract

**Objective:**

Multi-drug resistance (MDR) has notably increased in community acquired uropathogens causing urinary tract infections (UTIs), predominantly *Escherichia coli*. Uropathogenic *E. coli* causes 80% of uncomplicated community acquired UTIs, particularly in pre-menopausal women. Considering this high prevalence and the potential to spread antimicrobial resistant genes, the current study was conducted to investigate the presence of clinically important strains of *E. coli* in Pakistani women having uncomplicated cystitis and pyelonephritis. Women belonging to low-income groups were exclusively included in the study. Seventy-four isolates from urine samples were processed, phylotyped, and screened for the presence of two Single Nucleotide Polymorphisms (SNPs) particularly associated with a clinically important clonal group A of *E. coli* (CgA) followed by antibiotic susceptibility testing and genome sequence analysis.

**Results:**

Phylogroup B2 was most prevalent in patients and 44% of isolates were positive for the presence of CgA specific SNPs in Fumarate hydratase and DNA gyrase subunit B genes. Antibiotic susceptibility testing showed widespread resistance to trimethoprim-sulfamethoxazole and extended-spectrum beta-lactamase production. The infection analysis revealed the phylogroup B2 to be more pathogenic as compared to the other groups. The genome sequence of *E. coli* strain U17 revealed genes encoding virulence, multidrug resistance, and host colonization mechanisms.

**Conclusions:**

Our research findings not only validate the significant occurrence of multidrug-resistant clonal group A *E. coli* (CgA) in premenopausal Pakistani women suffering from cystitis and pyelonephritis but also reveal the presence of genes associated withvirulence, and drug efflux pumps. The detection of highly pathogenic, antimicrobial-resistant phylogroup B2 and CgA *E. coli* strains is likely to help in understanding the epidemiology of the pathogen and may ultimately help to reduce the impact of these strains on human health. Furthermore, the findings of this study will particularly help to reduce the prevalence of uncomplicated UTIs and the cost associated with their treatment in women belonging to low-income groups.

**Supplementary Information:**

The online version contains supplementary material available at 10.1186/s12866-024-03221-8.

## Introduction

The pattern of antibiotic resistance is different throughout the world depending on genetic variations in strains, and differences in the availability and frequency of utilization of antibiotics [1]. Despite these differences, it has been observed worldwide that antimicrobial resistance (AMR) and multi-drug resistance (MDR) has increased substantially among community-acquired uropathogens that cause urinary tract infections (UTIs), limiting the availability of treatment options utilizing oral antibiotics [2]. Modeling of uropathogens surveillance data collected in the United States from 2011 to 2019 demonstrates a relative average yearly increase of 2.7% drug-resistant phenotypes [3].

Uropathogenic *Escherichia coli* (UPEC) are the predominant pathogens causing community acquired UTIs (> 80%) and nosocomial UTIs (> 30%) [4]. Unique virulence profile and other genotypic characteristics of UPEC not only primarily link it to the occurrence of UTIs but to other extraintestinal infections as well [5]. Studies have also shown its potential association with the occurrence of gastrointestinal infections [6]. UTIs are conventionally classified on the basis of the site of infection, clinical symptoms of patients, microbiological laboratory findings and severity of disease. The major types are uncomplicated, complicated UTIs and urosepsis which are further categorized as upper and lower urinary tract infections [7]. Uncomplicated UTIs are usually more prevalent in healthy and adult non-pregnant women, whereas complicated UTIs (cUTIs) are not gender specific and could occur in all age groups. cUTI is often linked with either functional or structural urinary tract anomalies [8]. A study exploring the 30 years of global burden of UTIs reported a higher number of UTI cases and incidence rate among women as compared to men at the global level [9].

Women are more prone to UTIs as compared to men primarily owing to their different urinary system anatomy [10]. *E. coli* is the main cause of UTIs in 80% of healthy women aged 18–39 years followed by *Staphylococcus saprophyticus* (15–20%) [11]. In pregnant and non-pregnant women, this natural tendency of acquiring UTIs could be aggravated by multiple behavioural and psychosocial factors [12–14]. A major factor is low socioeconomic status (Low-SES) which in turn has been associated with multiple elements posing a risk to women’s health. Malnourishment causing weak immune system, poor hygiene and sanitation facilities [15–18], low level of knowledge and awareness, inaccessibility and unaffordability of basic health facilities, etc. have been associated with Low-SES. The importance of UTIs in the domain of public health could be gauged based on the fact that these infections are a common source of morbidity and have been reported to be associated with increased health care costs [19, 20]. The cumulative healthcare cost for the diagnosis and management of UTIs is estimated to be between 1.6 and 2.14 billion annually in the United States alone [11, 21].

*E. coli* isolates are grouped in different phylotypes or phylogroups which are defined on the basis of their ecological niche, life history traits, ability to cause disease, phenotypic and genotypic characteristics [22]. Furthermore, *E. coli* isolates having same sequence type are placed in different clonal groups [23].

Virulent extraintestinal *E. coli* strains including UPEC are mainly associated with phylogroups B2 and D [24–26] and a clinically important clonal group of *E. coli* i.e. clonal group A (CgA) was initially reported to belong exclusively to phylogroup D [27]. Isolates belonging to this clonal group were originally isolated from women having acute pyelonephritis and cystitis [28]. Although this clonal group is majorly associated with community acquired UTIs, its role as an etiological agent of hospital associated UTIs and various other extraintestinal infections has also been identified [29]. The distinctive virulence profile [27], high antibiotic resistance [27, 30] and relatedness with diarrheagenic *E. coli* (DEC) [6, 31] signifies the importance of this clonal group.

In this study, we aim to understand the distribution of *E. coli* phylogroups associated with uncomplicated cystitis and pyelonephritis in premenopausal women belonging to the low socioeconomic group of Pakistan. For this purpose, we initially determined and compared the predominant *E. coli* phylogroups associated with cases of uncomplicated UTIs. Subsequently, CgA status of the *E. coli* isolates was analyzed. We also investigated the virulence potential of different phylogroups by using invertebrate *Galleria mellonella* as an infection model system. In order to determine the factors associated with UTIs, we sequenced one of the highly virulent representative members of B2 phylogroup (identified by using *G. mellonella* infection model) and analyzed it vis-à-vis its pathogenesis and genome structure for this study.

## Materials and methods

### Study population

Urine samples were collected from female patients suffering from two specific classes of UTIs i.e., community acquired uncomplicated cystitis and pyelonephritis during the period from November 2016 to January 2017. Study participants were selected on the basis of following criteria:

#### Inclusion criteria

Premenopausal non-pregnant women (aged 18–49) with prior diagnosis of acute uncomplicated cystitis and pyelonephritis were approached. Their medical records were consulted for demographic and clinical information. The diagnosis was also verified using the criteria given by the European Association of Urology Section of Infection in Urology classification of UTIs based on clinical presentation, risk factors, and severity scale [32]. Regardless of their urban or rural background, samples were exclusively collected from women belonging to low socioeconomic group. The socioeconomic status scale developed by Kuppuswamy was used to define the socioeconomic status of the study population in the community [33].

#### Exclusion criteria

Women having pregnancy, menstruation, menopause, complicated UTIs, underlying diseases, prior antibiotic therapy and middle and upper socioeconomic status were excluded from the study.

#### Study setting

Major government tertiary care hospitals of three contiguously located cities (Islamabad, Rawalpindi and Taxila) were chosen on the basis of influx of diverse and large population seeking health care from several districts of Pakistan. The selected hospitals are also mainly frequented by population belonging to low and middle socioeconomic groups.

### Sample collection

Samples were collected after approval by ethical review boards of COMSATS University Islamabad and hospitals included in the study. Written consent was also taken from women fulfilling the inclusion criteria. Midstream urine samples were collected in a screw capped pre-sterilized 100 ml polypropylene container without any additives from women included in the study. To avoid any contamination or decline in microbe’s number, samples were carried to the microbiology laboratory of COMSATS University Islamabad, Islamabad Campus without delay and were cultured within 2 h for laboratory identification.

### Isolation and identification of *E. coli*

Samples were cultured on MacConkey agar (Oxoid, UK). A single suspected colony of *E. coli* was picked from the mixed cultures present on the surface of media and re-streaked on MacConkey agar plates and was further subjected to incubation at 37 °C overnight to get pure growth. Purified bacteria were examined by Gram staining and IMViC (Indole, Methyl Red and Voges-Proskauer (MRVP) and Citrate) utilization test indicating Indole positive, Methyl Red positive, Voges-Proskauer and Citrate negative for *E. coli* along with Lactose fermentation test [26, 34].

### DNA extraction and phylotyping

DNA of *E. coli* isolates was extracted by using the Phenol-Chloroform method [35]. The process of allocation of *E. coli* isolates to specific phylogroups has evolved over the last few decades [35–39]. Most recently a widely accepted technique for exclusive phylotyping of *E. coli* i.e., Clermont’s Triplex PCR, has been thoroughly revised [40]. The Quadruplex PCR not only places *E. coli* isolates into eight phylogroups i.e., A, B1, B2, C, D, E, F and Cryptic clade I but also gives an additional advantage to identify other cryptic clades and two more species of *Escherichia* isolates [41]. Therefore, phylogenetic analysis of all *E. coli* isolates was carried out by using Quadruplex PCR. Four sets of primers were used to detect the eight phylogroups, other possible cryptic clades and species of *Escherichia* (Table S2). PCR amplifications were done in 25 µl reaction mixture containing 2 µl DNA template, 0.75 µl of each primer, 3 µl of 10X Taq Buffer with (NH_4_)_2_SO_4_, 3.2 µl MgCl_2_, 0.5 µl of dNTPs and 0.3 µl Taq polymerase (Fermentas, Germany). Thermal cycling was performed in Bio-Rad MJ mini using the following conditions: 94 °C for 5 min; 30 cycles of denaturation at 94 °C for 1 min; annealing at 58 °C for 1 min and initial extension at 72 °C for 2 min. A final extension of 72 °C was run for 5 min. Duplex PCR was performed to differentiate D and E groups by using the primers initially used by Clermont and colleagues (2013) [41]. The conditions used for this PCR were the same as mentioned above.

### CgA screening of *E. coli* isolates

*Escherichia coli* clonal group A (CgA) causes disease in humans. CgA screening was done by subjecting phylogroups B2, D, E and F to SNPs (*fumC* and *gyrB*) detection via PCR [42]. Reference strains and primers for CgA specific PCRs were kindly provided by Statens Serum Institute, Denmark.

### fumC SNPs detection

Two positive (*E. coli* SE80003 and *E. coli* 3682) and one negative control (*E. coli* K5-23) were used in *fumC* SNPs detection PCR. Amplification of *fumC* was performed by singleplex PCR using one set of primers. PCR was run with a 25 µl reaction mixture containing 2 µl DNA template, 1 µl of a set of primers), 2.7 µl of 10X Taq Buffer with (NH_4_)_2_SO_4_, 3.7 µl MgCl_2_, 0.5 µl of dNTPs and 0.3 µl Taq polymerase (Fermentas, Germany).

The PCR conditions used were as follows: 95 °C for 5 min; 30 cycles of denaturation at 94 °C for 1 min; annealing at 55 °C for 1 min and initial extension at 68 °C for 3 min. A final extension of 72 °C was run for 10 min.

### gyrB SNPs detection

One positive (*E. coli* SE80003) and one negative control (*E. coli* F25988) were used in *gyrB* SNPs detection PCR. Amplification of *gyrB* was performed by singleplex PCR using one set of primers [42, 43]. PCR amplifications were done in 25 µl reaction mixture containing 2 µl DNA template, 1.5 µl of a set of primer, 2.5 µl 10X Taq Buffer with (NH_4_)_2_SO_4_, 3.7 µl MgCl_2_, 0.5 µl of dNTPs and 0.3 µl Taq polymerase (Fermentas, Germany). The PCR conditions used were as follows: 95 °C for 5 min; 30 cycles of denaturation at 94 °C for 1 min; annealing at 58 °C for 1 min and initial extension at 68 °C for 3 min. A final extension of 72 °C was run for 10 min. PCR reagents from Thermo Scientific, Fermentas were used for all sets of PCRs performed in the study.

### Antibiotic susceptibility testing of *E. coli* isolates

Antibiotic susceptibility testing of the isolates was carried out using modified Kirby-Bauer disk diffusion method on Muller-Hinton agar following the Clinical and Laboratory Standards Institute (CLSI) guidelines 2018 (CLSI document M07-A11) [44, 45]. *Escherichia coli* ATCC® 25,922 was used as the reference strain. The antibiotic disks were obtained from Oxoid, England. A lawn of microbes was made on Muller-Hinton plates by spreading the stock solution of the isolate that was prepared by dissolving 3–4 colonies into 500 µl PBS (Oxoid, UK) solution. After around 20 min, using sterile forceps, the appropriate antimicrobial disks were placed on the agar surface. Appropriate distance was maintained between the disks. Following antibiotics with mentioned concentrations were tested: Trimethoprim-sulfamethoxazole (STX 25 µg), Ceftazidime (CAZ 30 µg), Cefotaxime (CTX 30 µg), Nalidixic acid (NA 30 µg) and Ciprofloxacin (CIP 5 µg).

### ESBL detection

Double-disk diffusion method was used to screen the isolates as per CLSI guidelines, 2018 [46] for screening of ESBL producing isolates. The CLSI recommended use of cefotaxime (30 µg) and ceftazidime (30 µg) disks was followed for phenotypic confirmation of the presence of ESBL in *E. coli* isolates. A disc of Augmentin (20 µg Amoxycillin + 10 µg CLA) was placed in the center of plate on the surface of Mueller Hinton Agar. Then discs of Ceftazidime (30 µg), Cefotaxime (30 µg) and Aztreonam (30 µg) were placed around it in such a manner that each disc was at a distance ranging between 15 and 20 mm from the Augmentin disc (centre to centre).

### *Galleria mellonella* infection assay for *E. coli*

*G. mellonella* infection assays were performed in a manner similar to Champion et al., (2009) [47]. The infection assay was performed using selected strains belonging to different *E. coli* phylogroups. Briefly, bacterial broth cultures were grown overnight at 37^o^C. 10 µl of the bacterial suspensions OD600 nm was adjusted to a corresponding range of 10^2^ CFU to 10^8^ CFU per ml of nutrient broth and were injected into the first right proleg of the larvae (10 larvae per dose). Data from 3 independent experiments was used to calculate the percentage killing at infective doses i.e. 10^2^ to 10^8^ CFU per ml. *G. mellonella* larvae were scored at 24, 48 and 72 h. The scoring considered larval survival, movements (as surrogate for disease progression, i.e. ability to turn over) and color. Melanisation scoring system adapted from Senior et al. [48] was used and number of larvae exhibiting score of 4 (i.e. diseased) were then used to depict % survival.

### Genome Sequencing and assembly

Based on above mentioned testing and screening, *E. coli* strain U17 was selected for genome sequencing and analysis. The strain was grown overnight in LB medium at 30 °C in shaker incubator. Bacterial DNA extraction was performed using genomic DNA isolation Kit (QIAGEN, Germany) according to the manufacturer’s instructions. DNA quantity and quality were analyzed through Nanodrop 2000 (Thermo Fisher Scientific, Germany). Sequence library was constructed using a TruSeq Nano DNA Kit (Illumina, Inc., San Diego, CA), according to the manufacturer’s protocol, and sequencing was performed in a MiSeq 2 × 250-bp run. RAST was used for genome annotation, prediction of rRNA, tRNA, coding genes and GC content compositions. Clusters of Orthologous Groups of proteins (COGs) were used for the classification of predicted genes pertinent to virulence, drug resistance, secretory systems, and other pathways. Using the *E. coli* strain K12 genome as bait sequence, the genes conferring virulence and drug resistance or belonging to secretory clusters (T1SS to T6SS) [49] were retrieved from the selected *E. coli* genome. This was followed by independent confirmation through nucleotide BLAST analysis at NCBI as well as BioEdit [50, 51]. The genes with query coverage higher than 70% and greater than 50% similarity were taken as homologs [52]. The bacterial secretion system was also explored on the basis of web-based resources i.e., T346Hunter and SecReT6 [53, 54].

## Results and discussion

The current study was conducted in continuation of our previously published work on *E. coli* induced UTIs as a risk factor for preterm births in Pakistan [55, 56]. Another rationale for selection of the study population and UPEC as particular variant of Extraintestinal pathogenic *Escherichia coli* (ExPEC) was its significant relevance with a highly drug resistant and virulent clonal group A of *E. coli* [27].

### Phylotyping of UTI inducing *E. coli* isolates indicate high prevalence of pathogenic phylogroups

**Fig. 1:**
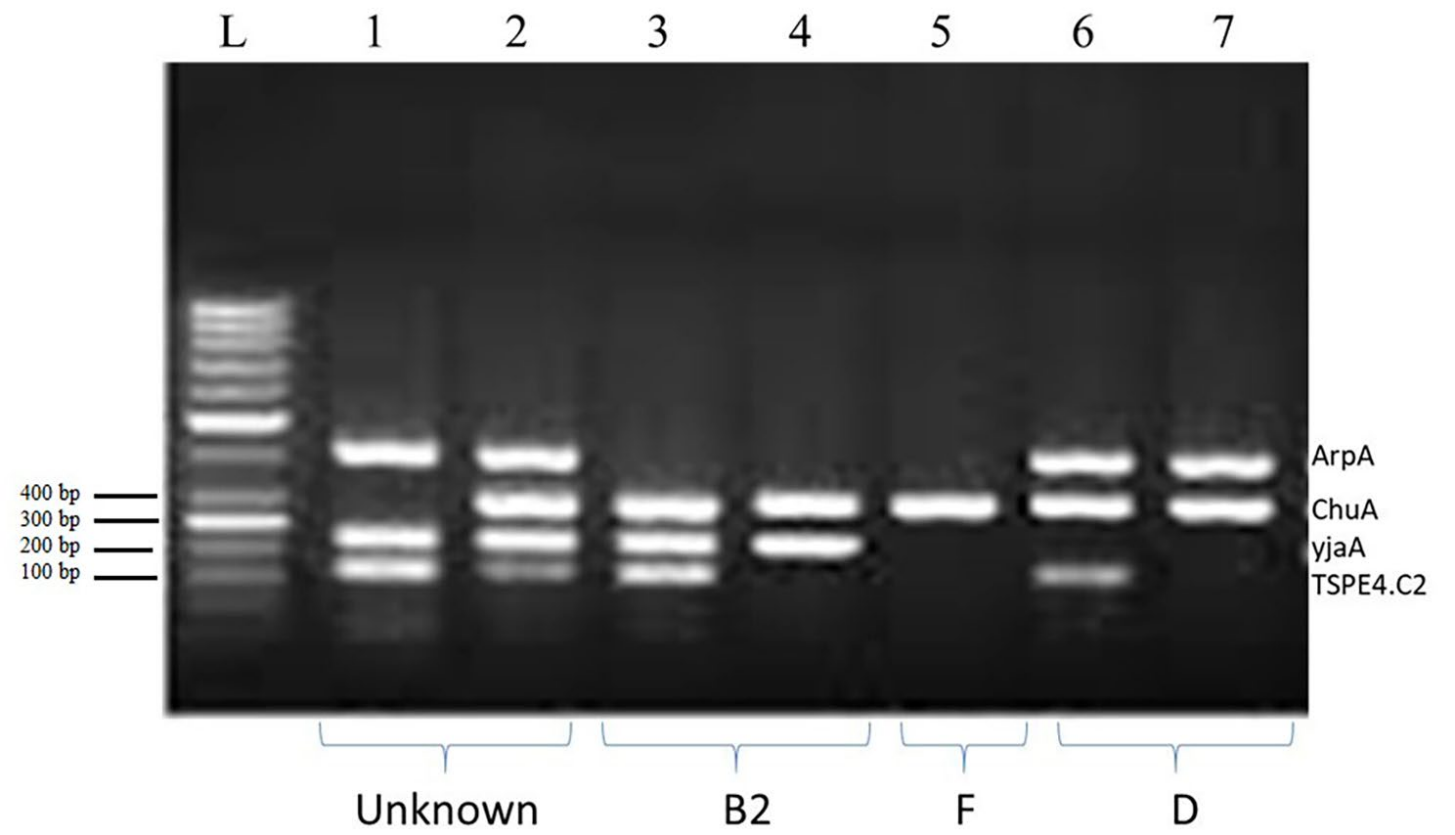
Phylogrouping of *E. coli* isolates. Representative gel electrogram indicating different phylotypes in lanes 1–7 electrophoresed on a 1.5% agarose gel

For the purpose of present study, *E. coli* isolates obtained from premenopausal women suffering from uncomplicated cystitis and pyelonephritis were systematically characterized using multiple techniques. Initially, UPEC isolates were subjected to phylotyping as it is an efficient way to establish virulence potential and common niche of *E. coli* isolates [22. Phylotyping of 74 UTI isolates revealed that B2 (34%) is the most prevalent group followed by D (18%) and E (12%). Other groups were detected in small proportion. Studies have shown that pathogenic *E. coli* strains causing extraintestinal infections mainly belong to group B2 and to a lesser extent to group D whereas commensal strains belong to group A and B1 [57]. Genotype of two unknown groups was also detected in a total of 13 isolates. Unknown group with a genotype of arpA-, chuA-, yjaA-, TSPE4.C2- (15%) was more prevalent (Table 1). Two isolates were found to have genotypes of an unknown group (arpA-, chuA-, yjaA+, TSPE4.C2+) and *E. albertii*. Eleven (15%) isolates that did not yield any band were analyzed using cryptic clade primers. However, these unknown isolates were confirmed belonging to *Escherichia* genus by using uidA and gadA/B primers [58]. The PCR results of this study are shown in Fig. 1.

**Table 1:** Determination of phylogroups and clonal group A status of 74 *E. coli* isolates

S#	Quadruplex Phylogroups	Number of Isolates	Number of Isolates belonging to Clonal group A ^b^
1	A	5	0
2	B1	3	0
3	B2	25	6
4	C	0	0
5	D	13	9
6	E	9	5
7	F	3	2
8	Cryptic Clade I	3	0
9	Cryptic Clade II, III, IV, V	0	0
10	Unknown groups ^a^		
	-,-,-,-	11	0
	-,-,+,+	1	0
	+,+,+,+	0	0
	+,-,+,+	0	0
11	*E. albertii*	1	0

^a^ Unknown groups are defined on the basis of the presence or absence of

*arpA*, *chuA*, *yjaA* and TSPE4.C2

^b^ No. of UPEC strains having *fumC* + *gyrB* SNPs

The prevalence of different *E. coli* phylogroups in UTI cases appears to be the same as reported in other previous studies [27, 30, 42, 55, 59]. A study from Iran reported high prevalence of phylogenetic group B2 (39.3%) followed by unknown (27.1%), E (9.3%), C and clade I (each 6.4%) whereas B1, F, D, and A groups were obtained in small numbers [59]. Subsequently, isolates belonging to phylogroups B2, D, E, and F were subjected to CgA screening. Although the number of studies based on the detection of CgA from other extraintestinal sites including diarrheal cases are limited, but they do suggest that CgA isolates are implicated in infections other than UTIs [30, 60]. CgA *E. coli* was obtained for the first time from women diagnosed with UTI. Around the world, the prevalence and characteristics of CgA isolates have mostly been studied in UTIs [27, 30, 42, 60, 61].

CgA screening revealed the presence of both fumC and gyrB SNPs in 22 isolates. Highest number of CgA isolates belonged to D phylogroup. The presence and distribution of both CgA specific SNPs among the four phylogroups is shown in Table 2. Interestingly, 32 CgA isolates were from patients having cystitis, whereas 12 CgA isolates were detected from samples gathered from patients having pyelonephritis suggesting that CgA isolates colonize preferred anatomical sites, as has been previously reported [62]. The worldwide prevalence of CgA isolates varies greatly as was observed in a multi-centered study conducted in representative countries and cities of six continents. In that survey, isolates from both urine and non-urine sources were included. Only 18 CgA isolates were reported from four countries of Asia whereas samples from India were negative for CgA isolates [55].

**Table 2:** Multidrug resistant UPECisolates belonging to different phylogroups

Isolate Phylogroup	MDR strains	Suspected ESBL	Common Resistant Antibiotic	Common Susceptible Antibiotic
A	2/5 (40%)	2/5 (40%)	SXT	NA, CAZ, CTX, CIP
B1	2/3(67%)	**-**	NA	SXT, CAZ, CTX, CIP
B2	21/25 (84%)	21/25 (84%)	SXT, NA, CAZ, CTX	CIP
C	-	**-**	-	-
D	11/13 (85%)	8/13 (61.5%)	SXT, NA, CTX, CIP	CAZ
E	6/9 (67%)	4/9 (44.4%)	SXT, CAZ, CIP	NA, CTX
F	2/3 (67%)	2/3 (66.66%)	SXT, NA, CAZ	CTX, CIP
Cryptic clade I	2/3 (67%)	2/3 (66.66%)	SXT, NA, CIP	CAZ, CTX
Unknown	3/12 (25%)	3/12 (25%)	SXT, NA, CAZ, CTX	CIP

### Prevalence of antimicrobial resistant uropathogenic *E. coli*

**Fig. 2:**
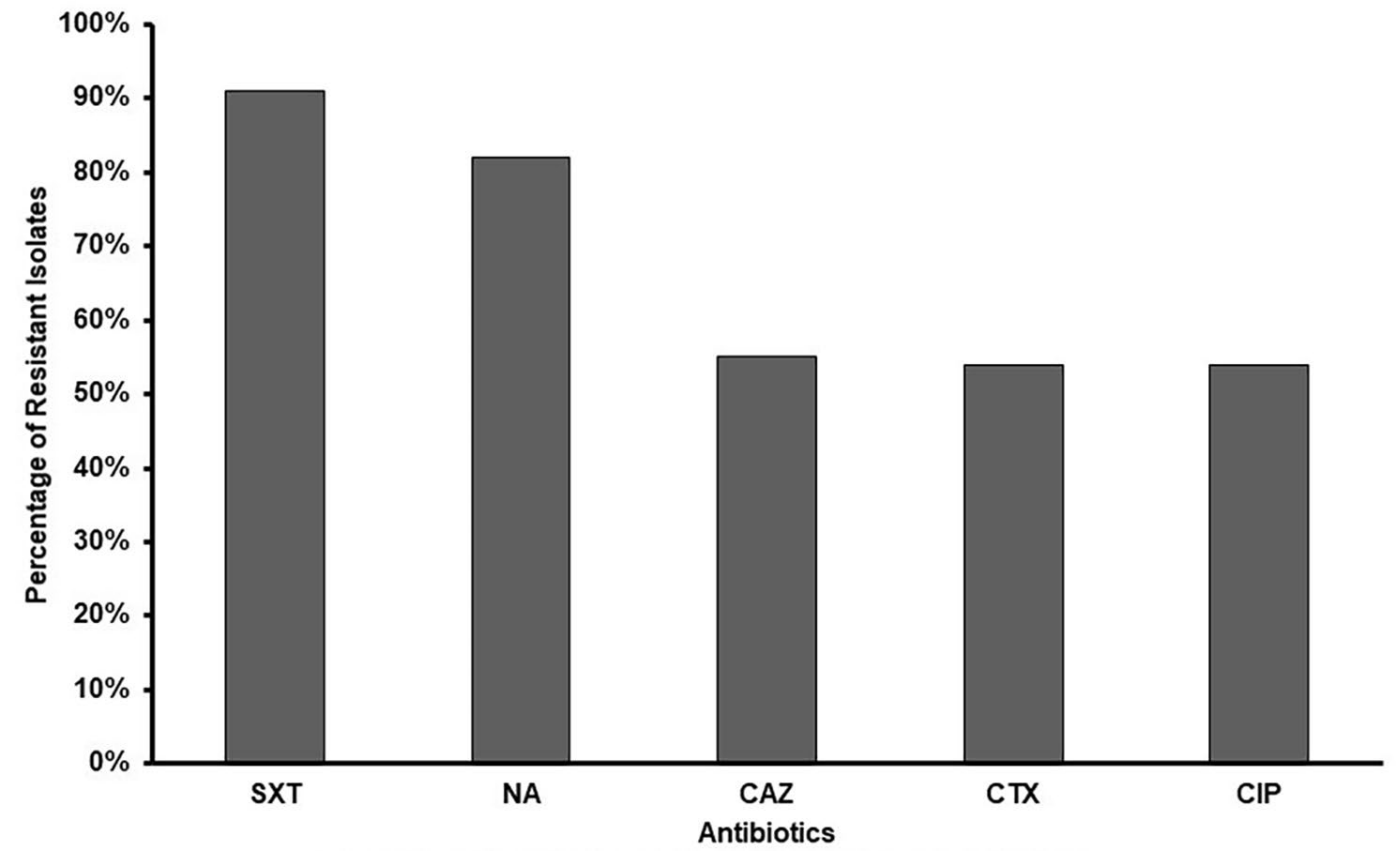
Antibiotic Susceptibility profiles of *E. coli* isolates obtained from females with UTIs

Rising antibiotic resistance among pathogenic *E. coli* is a cause of great concern for public health professionals [2]. Furthermore, evidence suggests that CgA *E. coli* isolates are highly drug resistant [63]. To observe the resistance pattern of uropathogenic *E. coli* isolates included in the present study, we tested their susceptibility to five antibiotics, which are commonly used for treating UTIs and other extraintestinal infections. The first-choice agents for treatment of uncomplicated UTIs in women include nitrofurantoin monohydrate/macrocrystals, and trimethoprim-sulfamethoxazole (Cotrimoxazole). Beta-lactam antibiotics may be prescribed when other recommended agents cannot be used [64]. The susceptibility tests conducted on all UPEC isolates indicated that these isolates exhibited resistance to nearly all the antibiotics examined in this study. Majority of the isolates were resistant to SXT (91%) followed by NA (82%) and other antibiotics (Fig. 2). The percentage of MDR strains belonging to B2 and D phylogroup was 84% and 84.6%, respectively. 67% of MDR strains belonged to B1, E, and F followed by A (40%) and Unknown groups (25%). In one study conducted in Pakistan, phylogenetic group B2 was predominant and a significant correlation between resistance to third-generation cephalosporins and ciprofloxacin was also observed [65]. Similarly, in the present study, the majority of the isolates belonged to B2 phylogroup and although the isolates were highly resistant to SXT [27, 63] and NA, the resistance to CTX, CAZ, and CIP was also notable as reported in a study from Iran [59].

Furthermore, 42 (57%) isolates were detected to be positive for ESBL (Table 2). It has also been confirmed in the present study that 19 (86%) out of 22 CgA *E. coli* isolates were SXT resistant and similar high resistance of CgA isolates to SXT has been reported in previous studies [27, 60, 63]. High prevalence of ESBL producers among uropahogenic *E. coli* isolates has also been reported in studies conducted in different parts of the world including Pakistan [66–69]. The prevalence of multidrug resistant *E. coli* in our study population could be attributed to the microbes’ ability to continuously acquire resistance against these drugs from the environment. However, detailed investigations are required to support this.

### Determination of *E. coli* pathogenicity using *G. mellonella* infection model

*G. mellonella* model has been recently used to investigate the pathogenesis of *E. coli* [70, 71]. However, the comparative analysis of pathogenic potentials of UTI associated *E. coli* isolates belonging to different phylogroups has not been previously studied using this model. Our results are in accordance with those reported by Ciesielczuk and colleagues [72] who reported that isolates of ST131 belonging to phylogenetic group B2 were also associated with high virulence.

**Fig. 3:**
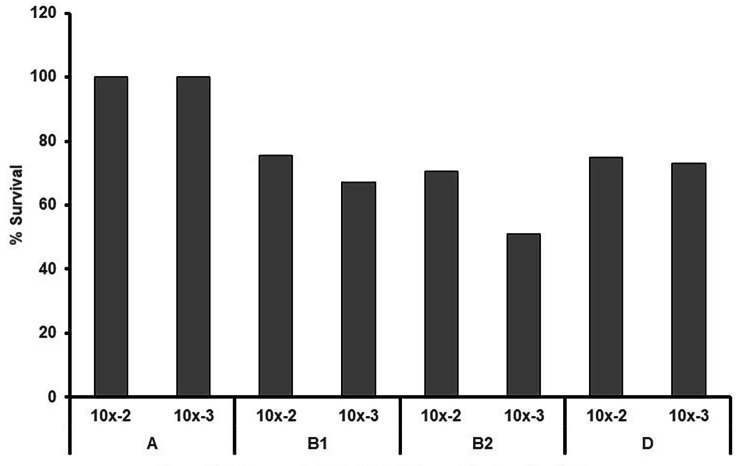
*G. mellonella* infection assay for *E. coli* phylotypes. Bacterial suspensions of overnight cultures corresponding to a range of 10^8^ CFU to 10^2^ CFU were injected into the first right proleg of the larvae. *G. melonella* larvae were scored at 72 h for: survival, movement (as surrogate for disease, i.e. ability to turn over), and colour

We have also shown that this simple invertebrate model was able to distinguish between the pathogenic potential of different phylotypes. It was observed that at high concentrations ranging upto 10^2^ and 10^3^ CFU/larva, the strains belonging to phylotype B2 and B1 respectively induced 50% and 35% killing or septic death in *Galleria* in a dose dependent manner. However, phylogroup A did not induce larval death, even at 10^3^ CFU/larva (Fig. 3). The enhanced killing ability of B2 group can be due to range of virulence factors/siderophores/iron chelating molecules such as yersiniabactin etc. [73, 74]. In this assay, we used *Galleria* killing as an end point to monitor the progress of infection. However, additional symptoms such as signs of melanization and changing body coloration, pupa formation was also observed at 72 h and our study suggested that the *G. mellonella* infection model is a simple, cheap and useful tool for assessing virulence of different clonal types of UPEC.

### Features of *Escherichia coli* phylotype B2 strain U17 genome

**Fig. 4:**
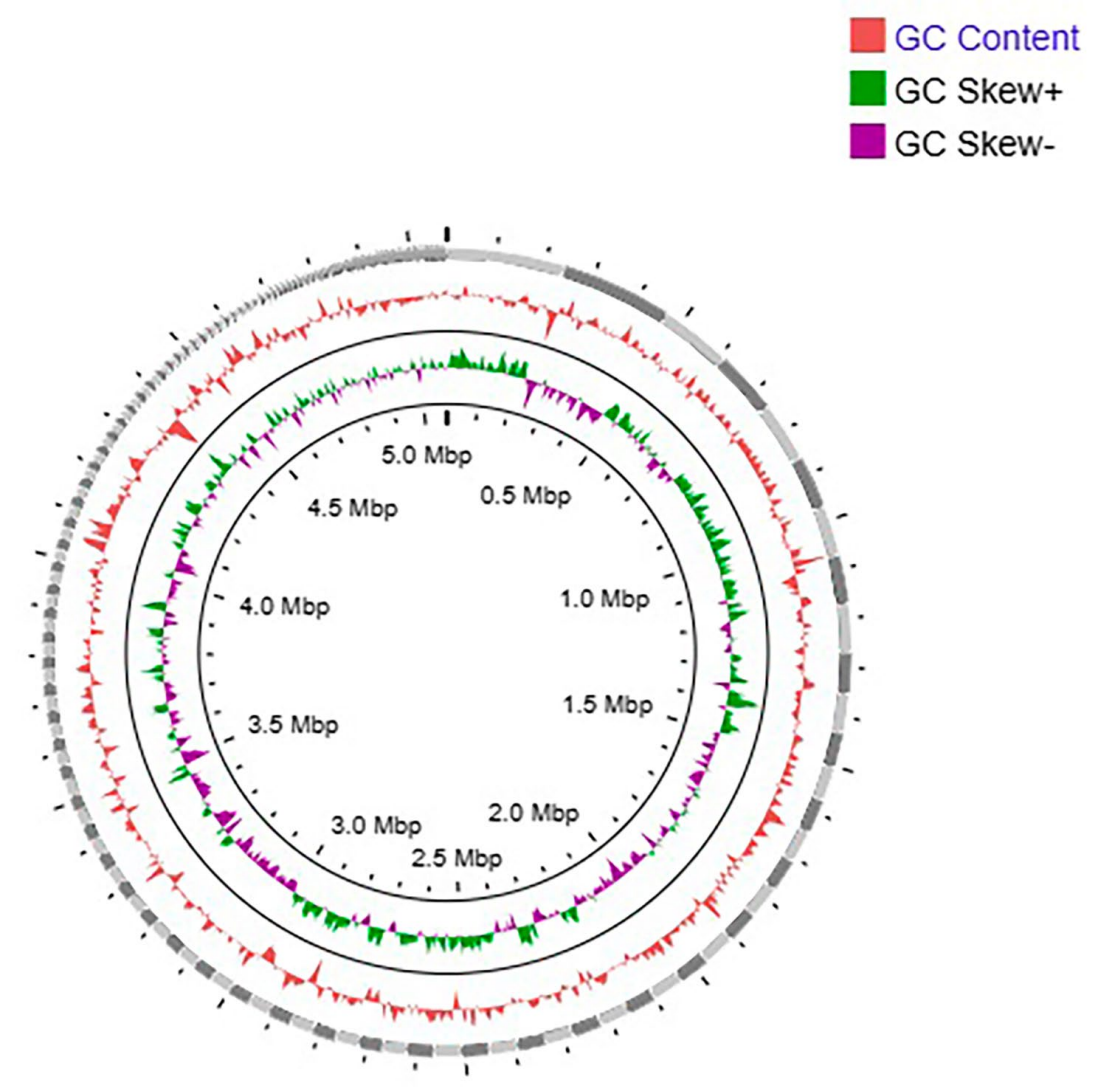
Graphical representation of *E. coli* genome with GC contents and GC skew

Analysis of the genome of an *E. coli* phylogroup B2 strain from this study was conducted in order to better characterize this highly prevalent and pathogenic phylogroup which could further help in understanding the pathogenesis and niche adaptation as well as their role in shaping up the microbiota of a specific site. Genome sequencing of phylogroup B2 strain U17 showed presence of a single circular chromosomal DNA of 5.05 Mbp having 493 contigs. The genetic information consists of 5,339 genes, exhibiting a GC content of 50.7%, and includes 86 RNAs (Table 3). The representation of the entire genome GC content is summarized in Fig. 4. The complete genome sequence of *E. coli* strain K12 which was used as a reference genome has in comparison a similar GC content (50.8%), while the number of coding sequences was 5,683. *E. coli* strains are abundantly present in nature and are also notorious in causing various diseases and contain many well characterized genes linked to pathogenesis, such as colonization, host adherence, and bacterial survival in the urinary tract [5].

**Table 3:** Genomic features of *E. coli* B2 strain U17 and K12 species analyzed in the study

Features	E. coli B2 U17	E. coli K12
Origin	Human	Human
Chromosome No.	493	3 scaffolds
Genome Size (Mb)	5.09	4.63
Coding Gene No.	5, 339	5, 683
G + C Content (%)	50.07	50.08
RNAs	86	88

Due to the presence of high multidrug resistance among our isolates, extensive genome computational analysis such as BLASTN was conducted for the genome survey of drug resistant genes in *E. coli*. Databases like Antibiotic resistance genes database (ARDB) [75] and bacterial VFDB (virulence factors database) [76] were also used to verify the drug resistant genes dataset and predicted virulence associated genes in *E. coli* genome sequence in our study. Our genome analysis revealed that *E. coli* harbors more than 70 various types of efflux genes such as putative manganese efflux pump MntP, Leucine efflux protein, multidrug efflux system MdtABC-TolC, membrane fusion component MdtA etc. (Table S1). Drug resistance in *E. coli* strains included in this study could be attributed to the presence of these efflux pumps [77]. Moreover, in our genome wide analysis, we identified carbapenemase associated genes which are β-lactamases with versatile hydrolytic capacities such as to hydrolyze penicillins, cephalosporins, monobactams, and carbapenems (Table S1). *E. coli* phylotype B2 strains producing these β-lactamases may cause serious recurrent infections.

Gram-negative bacteria possess various specialized secretory apparatus. Some of these secretory systems such as the type VI secretion system (T6SS) deliver effector proteins into host cells (either eukaryotes or prokaryotes) in a contact-dependent manner. Certain T6SS effectors exert anti-prokaryotic or anti-eukaryotic activity by targeting the cell wall, membrane or the nucleic acid [78]. This system comprises of at least 14 subunits forming the core machinery apparatus which is also called the imp operon. Further research should focus on exploring potential approaches by targeting T6SS effectors so as to facilitate the development of effective antibacterial drugs to treat UTIs and other diseases caused by ExPEC.

However, presence of molecular features existing in both enteroaggregative and uropathogenic *E. coli* strains have been shown in the same isolate, which endorse the hypothesis that several other genetic determinants play an important role in bacterial persistence in different niches. The genome wide analysis of *E. coli* revealed the presence of entire Imps operon in three loci, as well as T6SS component protein, and Hemolysin coregulated protein (Hcp) as an orphan component in the U17 strain studied here (Table S2).

The T6SS is a nanomachine for protein translocation found extensively in Gram-negative bacteria. It functions as a mechanism to transport effectors directly into the cells of target bacteria or eukaryotes [79]. T6SS is versatile as it can impact bacterial virulence, as well as competitiveness. The intracellular multiplication protein F (icmf) family protein possesses ATPase activity energizing the T6SS system. However, the absence of this protein in *E. coli* may indicate a non-functional T6SS system, or these species may adopt other pathways. Our comparative genomic analyses indicated that an integrated T6SS gene cluster probably could be the main player participating and facilitating bacterial adherence and invasion to host cells.

The identification of *E. coli* isolates associated with the highly virulent and antibiotic-resistant clonal group A in the study population chosen for this research has not been previously documented in Pakistan, nor has it been comprehensively addressed in previous studies within the South-Asian region. Many countries in this area fall within the lower-middle income category, leading to an overwhelming strain on healthcare systems and making it challenging for patients to manage healthcare expenses. With these factors in mind, our research specifically focused on women from disadvantaged socioeconomic backgrounds, who are more susceptible to various infections that can be prevented through simple changes in behavior. These behavioral changes include but are not limited to the acquisition of knowledge regarding the prevention and treatment of uncomplicated UTIs and the adoption of proper nutritional and hygienic practices. The findings from this study have the potential to significantly reduce the prevalence of uncomplicated UTIs and decrease the resistance to antibiotics commonly used for their treatment.

## Conclusions

Our study shows that the phylogroup B2 is most prevalent in premenopausal women suffering from uncomplicated UTIs. The existence of multidrug resistant and ESBL producing CgA *E. coli* isolates in premenopausal women of South Asian region suffering from uncomplicated UTIs has been observed as well. The higher invasive ability of the B2 phylogroup was confirmed using *G. mellonella* infection model indicating that infections with these strains pose a greater risk for uncomplicated UTIs. CgA isolates may have potentially posed a greater threat at such clinical sites by expressing the range of virulence factors. It would be worth investigating its nosocomial and community transmission patterns further enhancing our understanding of its environmental niches for better control. Extensive antimicrobial resistance of uropathogenic *E. coli* and CgA isolates necessitate the adoption of alternate strategies to circumvent antibiotic resistance while treating cystitis and pyelonephritis associated with such isolates. The high prevalence of extensive antimicrobial resistance in strain U17, as evidenced by the identification of over 70 drug and virulence resistance genes in its genome, highlights its pronounced infectious potential. It could further be assumed that the prevalence of UTIs due to virulent strains of *E. coli* in our study might be due to risk factors associated with low income group. This could be further validated by a detailed analysis of behavioral, clinical, and psychosocial risk factors attributed to women of different socioeconomic groups in Pakistan.

### Electronic supplementary material

Below is the link to the electronic supplementary material.

Supplementary Material 1

Supplementary Material 2

Supplementary Material 3

Supplementary Material 4

Supplementary Material 5

## Data Availability

This Whole Genome Shotgun project has been deposited at DDBJ/ENA/GenBank under the accession JABFHH000000000. The version described in this paper is version JABFHH010000000.
