# Supplementary material for: Multidrug resistance among uropathogenic clonal group A E. Coli isolates from Pakistani women with uncomplicated urinary tract infections
Source: BMC Microbiol. 2024 Mar 7;24:74. doi: 10.1186/s12866-024-03221-8 (PMC10919050; doi:10.1186/s12866-024-03221-8)
Supplement: Supplementary file 1 — Supplementary Material 1 [file 12866_2024_3221_MOESM1_ESM.docx]

**Multidrug Resistance Among Uropathogenic Clonal Group A *E. coli* isolates from Pakistani Women with uncomplicated urinary tract infections**

Ayesha Khan^1,2¶^, Viqar Sayeed Saraf^1¶^, Fariha Siddiqui^3^, Tahira Batool^1^, Zobia Noreen^1^, Sundus Javed^1^, Aftab Ahmed^4^, Wadi B Alonazi^5^, Muhammad Ibrahim^6^, Sandra Pucciarelli^7^, Habib Bokhari^4^*

^1^Microbiology and Public Health Lab, Department of Biosciences, COMSATS University Islamabad, Islamabad, Pakistan

^2^Health Services Academy, NIH Opposite, Islamabad, Pakistan

^3^Department of Biosciences, Shifa Tameer e Millat University, Islamabad, Pakistan.

^4^Department of Microbiology, Kohsar University Murree, Rawalpindi, Pakistan

^5^Health Administration Department, College of Business Administration, King Saud University, Riyadh, Saudi Arabia

^6^Department of Biosciences, COMSATS University Islamabad, Sahiwal Campus, Pakistan

^7^School of Biosciences and Veterinary Medicine, University of Camerino, via Gentile III da Varano, 62032 Camerino, Italy

**Running Title:** Urinary tract infections in premenopausal women of low income group

^¶^ Authors with equal contribution

*** Corresponding author:** Prof. Dr**.** Habib Bokhari**/**Dr. Muhammad Ibrahim

Department of Microbiology, Kohsar University Murree, Punjab, Pakistan

Email: [vc@kum.edu.pk](mailto:vc@kum.edu.pk)/ibrahim@cuisahiwal.edu.pk

**Supplementary Data**

**Table S1.** Phylogrouping of *E. coli* by their respective genes combinations obtained by Quadruplex PCR

**Table S2.** Computational prediction and characterizations of antibiotic resistance and multidrug resistance components in the *Escherichia coli* Phylotype B2 strain U17 and K-12

**Table S3.** Computational prediction and characterizations of T6SS components in the genus *Escherichia coli* Phylotype B2 strain U17 with K-12

**Supplementary Figure 1:** Gel images showing amplified products of Clonal group A specific genes. a) fumC. Lane 1: 100bp Ladder; Lane 2, 4, 5 and 8 shows the positive results. Lane 13 and 14: 1+ve = *E. coli* SE8003 and 2+ ve = *E. coli* 3682; Lane 15: -ve = *E. coli* K5-23 b) gyrB. Lane 1: 100bp Ladder; Lane 2 to 13 shows the positive results. Lane 14: + ve ctrl = *E. coli* SE80003.
