# Supplementary figures and images for: Multidrug resistance among uropathogenic clonal group A E. Coli isolates from Pakistani women with uncomplicated urinary tract infections
Source: BMC Microbiol. 2024 Mar 7;24:74. doi: 10.1186/s12866-024-03221-8 (PMC10919050; doi:10.1186/s12866-024-03221-8)

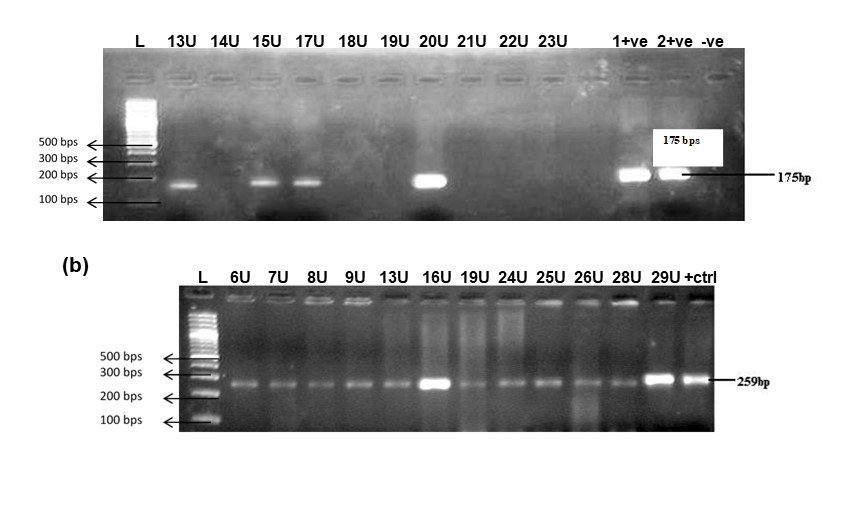

Supplement: Supplementary file 2 — Supplementary Material 2 [file 12866_2024_3221_MOESM2_ESM.jpg]
