# Supplementary material for: Multidrug resistance among uropathogenic clonal group A E. Coli isolates from Pakistani women with uncomplicated urinary tract infections
Source: BMC Microbiol. 2024 Mar 7;24:74. doi: 10.1186/s12866-024-03221-8 (PMC10919050; doi:10.1186/s12866-024-03221-8)
Supplement: Supplementary file 3 — Supplementary Material 3 [file 12866_2024_3221_MOESM3_ESM.docx]

**Table S1.** Phylogrouping of *E. coli* by their respective genes combinations obtained by Quadruplex PCR

| **Amplicons** | **ArpA= 400bp, ChuA=288bp, yjaA=211bp, TSPE4.C2=152bp** |
| --- | --- |
| **Phylogroups** | **Genes Distribution in Different Groups** |
| **A** | ArpA **or** (ArpA + yjaA) |
| **B1** | (ArpA + TSPE4.C2) |
| **B2** | (ChuA + yjaA) **or** (ChuA + TSPE4.C2) **or** (ChuA + yjaA + TSPE4.C2) |
| **C** | (ArpA + yjaA) |
| **D** | (ArpA + ChuA + TSPE4.C2) **or** (ArpA + ChuA) |
| **E** | (ArpA + ChuA) **or**  (ArpA + ChuA + yjaA) **or** (ArpA + ChuA + TSPE4.C2) |
| **F** | ChuA |
